# The Role of Association in Early Word-Learning

**DOI:** 10.3389/fpsyg.2012.00283

**Published:** 2012-08-21

**Authors:** Gary F. Marcus, Keith J. Fernandes, Scott P. Johnson

**Affiliations:** ^1^Department of Psychology, New York UniversityNew York, NY, USA; ^2^Department of Psychology, University of California Los AngelesLos Angeles, CA, USA

**Keywords:** word-learning, association, cognitive development

## Abstract

Word-learning likely involves a multiplicity of components, some domain-general, others domain-specific. Against the background of recent studies that suggest that word-learning is domain-specific, we investigated the associative component of word-learning. Seven- and 14-month-old infants viewed a pair of events in which a monkey or a truck moved back and forth, accompanied by a sung syllable or a tone, matched for pitch. Following habituation, infants were presented with displays in which the visual-auditory pairings were preserved or switched, and looked longer at the “switch” events when exposure time was sufficient to learn the intermodal association. At 7 months, performance on speech and tones conditions was statistically identical; at 14 months, infants had begun to favor speech. Thus, the associative component of word-learning does not appear (in contrast to rule-learning, Marcus et al., 2007) to initially privilege speech.

## Introduction

Word-learning consists of a mapping between an auditory signal or visual sign and an object or event (Brown, 1958; Hirsh-Pasek and Golinkoff, 1996). For hearing infants, word-learning is primarily an intermodal mapping of units of speech, produced by other people, and referents, such as objects, properties, and actions. Is speech a necessary component of this mapping, or could any sound do?

Considerable effort has been devoted to this question, but thus far results have been mixed. One substantial contingent of word-learning studies has revealed an advantage for speech relative to non-linguistic stimuli, but another has seemingly shown no such advantage for speech relative to stimuli such as tones or gestures. The picture is complex, as extant studies differ both in the ages in which subjects were tested, and the methodologies that were used.

## An Advantage for Speech

Among studies showing advantage for speech are Namy and Waxman (1998), who found that in a word-learning task, 26-month-olds (though not 18-month-olds) accepted words but not gestures as labels for novel categories, and Woodward and Hoyne (1999) who found an advantage for speech relative to non-linguistic beeps and squeaks at 20 months but not at 13 months in a multiple-choice word-learning task. Similarly Balaban and Waxman (1997) found that 9-month-olds performed better on a categorization task when test items were paired with words than when they were paired with tones. Infants were familiarized with slides of animals (e.g., rabbits) as they heard word phrases or tones, followed by a test phase in which two new animals were presented, one from within the category (e.g., a new rabbit) and one from outside it (e.g., a pig). Infants who heard the word phrase looked longer at the animal outside the familiarization category, interpreted as a novelty preference, but infants who heard the tone showed no preference. This result was recently replicated in younger children (6-month-olds by Fulkerson and Waxman, 2007; 3- and 4-month-olds by Ferry et al., 2010). In a different context, object individuation, Xu (2002), reported that two unique labels, presented simultaneously with objects brought out individually from behind an occluding screen, facilitated 9-month-olds’ establishment of representations of two unique objects. Other sounds – tones, toys, or emotional expressions – did not. More broadly, across the full range of studies, the clearest evidence for an advantage for speech comes from word-learning tasks, with older children, but advantages for speech have also been found in other tasks (e.g., categorization) with younger children, and have been absent even with older children in other tasks (e.g., Namy, 2001).

## No Advantage for Speech

On the other hand, in a categorization task somewhat similar to word-learning tasks described previously, Roberts (1995) reported evidence that 15-month-olds formed appropriate object categories when the presentation of auditory input, either speech or music, was synchronized with infants’ visual attention to the stimuli. In another object categorization task Namy (2001) found that 17-month-olds are equally open to accepting words, gestures, musical sounds, or pictograms as labels for object categories. Likewise, in an object individuation paradigm with younger children than those observed by Xu (2002). Wilcox et al. (2006) found that alternating presentation of two different rattle sounds (uncooked rice and small bells) led 4.5-month-olds to look longer at a one-object display when revealed from behind an occluder vs. two identical objects, implying that infants expected two rather than one, presumably on the basis of the two non-linguistic sounds. Thus two different sounds supported infants’ inference of two objects that are otherwise identical, though there was no test of whether infants made associations between the individual sounds and each object. Whether this difference is driven by age or methodological differences is not yet clear.

## The Current Study

No single study is likely to resolve this complex pattern of results, in part because there are many differences in stimuli, methods, and in the ages of children tested. A theoretical perspective that might yield some traction is the notion that word-learning is not a single unified module, but rather an accumulation of disparate mechanisms, some (Marcus and Rabagliati, 2006) or all of which may be domain-general (Bloom, 2000)^1^. In an effort to begin to decompose word-learning into constituent parts, we focus here on a single aspect of word-learning – the drawing of associations between a label and its referent, in simple and direct circumstances (see Waxman and Gelman, 2009 and Sloutsky, 2003 for discussions about the overall role of association in word-learning), while at the same time limiting the contributions of rich referential task. (In this respect, our strategy is the opposite of studies such as Namy, 2001, which explored situations in which the referential context was deliberately rich.) Although prior work has made it clear that young infants can draw arbitrary cross-modal associations (e.g., Slater et al., 1997, 1999) less is known about their ability to draw associations in the service of word-learning.

We investigated word-learning in two age groups with a cross-sectional design. The younger age group was 7 months of age, a point in development at which biases might be attributable not to extensive familiarity with a particular language (e.g., Werker and Tees, 1984) but rather with whatever prior knowledge children might have about language. Previous work has shown that 7-month-olds privilege speech relative to non-linguistic materials such as musical tones (Marcus et al., 2007), and that infants (and even newborns) prefer listening to speech relative to closely matched non-speech controls (Vouloumanos and Werker, 2004, 2007). Here we ask whether children of a similar age privilege speech in a word-learning task that emphasizes the associative component of word-learning.

We also investigated learning of visual-auditory associations in an older sample of children, 14-month-olds. One might imagine that as children acquired a larger vocabulary, they might eventually come to privilege speech in even a word-learning situation that was primarily associative in nature. Alternatively, the associative component might remain neutral with respect to content even as a child established a larger vocabulary.

To provide the most stringent test possible of the potential facilitative effects of speech vs. non-speech sounds in associative learning, we modeled our study upon the methods of Gogate and Bahrick, 1998; cf. Balaban and Waxman, 1997), who have shown that infants at 7 months can learn arbitrary associations between speech sounds and events in which an object is shown moving back and forth. Gogate and Bahrick investigated the ability to relate vowel sounds with objects when intersensory redundancy was present vs. absent. Infants were habituated to two alternating videos of vowel-object pairs in which the movement of an object was temporally coordinated with a spoken sound, a scenario which, the authors reasoned, simulates showing and naming the objects to the infant. Infants were reported to detect a mismatch in the vowel-object pairs in a synchronous condition, but not an asynchronous condition. Because we wished to illuminate differences between speech vs. non-speech in association learning, however, rather than effects of synchronous vs. asynchronous movement, we elected to present all events as asynchronous. A pilot experiment, described in more detail subsequently, provided evidence of association learning in many infants even under these more demanding conditions.

## Materials and Methods

### Participants

The final sample consisted of 36 full-term 7-month-old infants (17 female, 19 male, *M* age = 221 days, range 201–246, SD = 15.4) and 36 full-term 14-month-old infants (15 female, 21 male, *M* age = 433 days, range 411–453, SD = 13.4). Eighteen additional infants were observed but excluded from the final sample due to fussiness (nine 7-month-olds and five 14-month-olds), insufficient attention (one 7-month-old and one 14-month-old), or experimenter error (one 7-month-old and one 14-month-old). Infants were recruited by letter and telephone from an established database of families. Parents and infants received a small gift (a toy or t-shirt) for their participation. The experimental protocol was approved by the Institutional Review Board.

### Apparatus and stimuli

A Macintosh G4 computer and 76 cm color monitor were used to present stimuli. Infants were seated approximately 120 cm from the screen. Two speakers were placed 70 cm apart just behind the front surface of the monitor. An experimenter viewed the infant over a closed-circuit television camera and coded looking times online by pressing a key when the infant was looking. The experimenter was blind to the stimulus being presented on screen.

**Figure 1:**
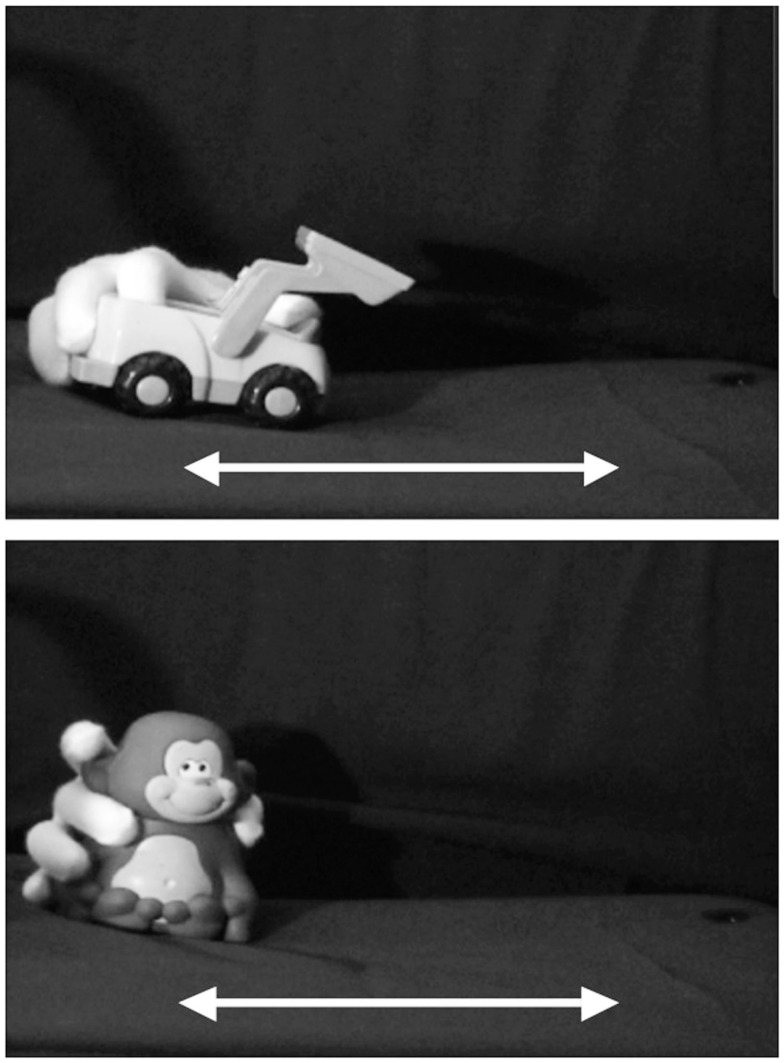
**Truck and monkey events**.

Two live visual events were recorded, each involving a gloved hand moving a toy (truck or monkey) back and forth horizontally (see Figure 1). Common, everyday objects were chosen so as to minimize computational/memory demands, allowing our investigation to focus as directly as possible on association *per se*.

Events took place on a table covered with black fabric, and black fabric was hung as a curtain to shield the arm and body of the actor who moved the toys. A complete cycle of motion took 4.0 s, and the toy moved continuously back and forth for the duration of each trial. The truck and monkey events were each accompanied by a sound that was played once during each translation of the toy (cf. Werker et al., 1998). The sound during each translation began at a random interval relative to commencement of translation that varied from 0 to 1000 ms, ensuring that visual and auditory onsets and offsets were asynchronous. The truck was yellow with a gray scoop and black wheels and measured 16.2 cm × 32.6 cm (9.8° × 19.5° visual angle at the infant’s 120 cm viewing distance), and the monkey was green and tan and measured 19.4 cm × 17.9 cm (11.7° × 10.8°). The horizontal path of motion measured 37.4 cm (22.4°).

Infants were randomly assigned to either the *Speech* condition, in which each visual event (truck or monkey) was accompanied by a speech syllable (*di* or *ga*), or the *Tones* condition, in which each visual event was accompanied by a piano note (F or Bb). This allows us to keep constant pitch while varying only the factor of interest, i.e., the presence or absence of phonetic detail; it also allowed us to avoid concerns that musicality itself might be distracting and hence impair performance. Our work finding that infants could learn rules from speech was originally done with synthetic speech (Marcus et al., 1999), but has since been shown to replicate with sequences of sung syllables (Marcus et al., 2007), yet not with tones. The stimuli used here therefore match the contrast of Marcus et al. (2007) between sung syllables and tones, but with the intriguingly different result that rule-learning, but not word association, significantly privileges sung syllables over tones. Speech stimuli were produced by a musically trained female vocalist singing the syllables *di* or *ga* at F and Bb, respectively, both in the octave that begins with middle C of a standard keyboard. Tones stimuli were computer-generated piano notes at the same pitches. Speech and tomes stimuli were 400 ms in duration. Which stimulus (monkey or truck) appeared first during habituation was determined randomly. For half of the infants, the truck was accompanied by *di* (Speech condition) or F played on the piano (Tones condition), and the monkey was accompanied by *ga* or Bb; for the other half of the infants, these pairings were reversed.

### Procedure

The experiment was prepared using Habit X software (Cohen et al., 2004). Each trial began with the presentation of an engaging attention-getter (an expanding and contracting ball that beeped in conjunction with its motion). When the experimenter determined that the infant was looking at the monitor, he or she pressed a key to initiate stimulus presentation. When the infant turned away from the monitor, the experimenter released the key. If the infant returned attention toward the screen the experimenter again pressed the key and the trial continued; otherwise trials were terminated after 2 s of continuous looking away or a maximum of 60 s.

The truck and monkey were presented in alternation until the infant reached a criterion of habituation, defined as a decline in looking times across two pairs of trials (i.e., four consecutive trials) of more than 50% of looking time during the first four trials. These were followed by four test trials. In two of these displays, the sound accompanying the visual event was identical to the pairings experienced during habituation (*Same* trials) and in the other two, the sound was switched (*Switch* trials). The four test trials were ordered pseudo-randomly, with one instance of same and switch trials in each of the two pairs.

## Results

**Figure 2:**
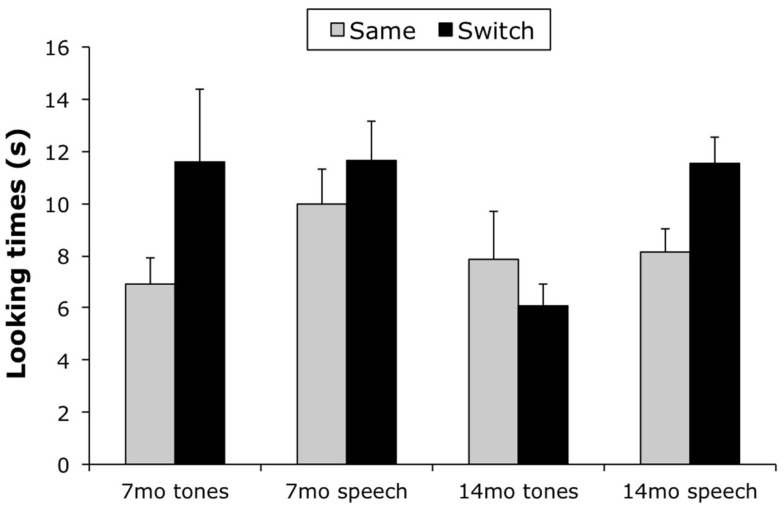
**Looking times for 7- and 14-month-olds in Tones and Speech conditions**. Looking times for 7-month-olds were significantly longer during Switch trials across conditions, but looking times for 14-month-olds were significantly longer during Switch trials only in the Speech condition.

The principal dependent measure was looking times at the two kinds of test displays. Longer looking during Switch trials would indicate that infants detected the association of the visual-auditory pairings presented during habituation, and looked longer due to their novelty relative to Same trials. Looking time data in many cells (both habituation and test) were positively skewed, violating assumptions of homogeneity of variance required by ANOVA; therefore data were log-transformed prior to analysis (raw scores are reported in the text and in Figure 2). Preliminary analyses incorporating order of habituation stimulus (truck vs. monkey first; *di*/*ga* vs. F/Bb first), order of test stimulus (same vs. switch), and sex of participant revealed no reliable effects that bore on our questions of association learning; therefore subsequent analyses were collapsed over these variables.

We tested for differences in habituation times between infants in the Speech and Tones conditions with a 2 (Condition: Speech vs. Tones) × 2 (Age Group) ANOVA and found longer looking overall by infants in the Speech condition, *F*(1, 68) = 8.89, *p* < 0.01, partial η^2^ = 0.12 (*M* for Speech = 210.8 s, SD = 101.6; *M* for Tones = 149.5 s, SD = 76.6), and longer looking overall by the younger infants, *F*(1, 68) 7.00, *p* < 0.05, partial η^2^ = 0.09. The interaction was not statistically significant.

Next, we tested for association learning with a 2 (Condition: Speech vs. Tones) × 2 (Age Group) × 2 (Association: Same vs. Switch) mixed ANOVA, with repeated measures on the third factor. There was a reliable main effect of Condition, *F*(1, 68) = 9.28, *p* < 0.01, partial η^2^ = 0.12, reflecting longer looking overall by infants in the Speech condition (*M* = 10.32 s, SD = 5.26) vs. the Tones condition (*M* = 8.13 s, SD = 7.68), and a reliable main effect of Association, *F*(1, 68) = 9.04, *p* < 0.01, partial η^2^ = 0.12, reflecting longer looking overall during Switch trials (*M* = 11.63 s, SD = 6.45) vs. Same trials (*M* = 9.99 s, SD = 5.76). There was also a reliable Condition × Age Group × Association interaction, *F*(1, 68) = 6.82, *p* < 0.05, partial η^2^ = 0.11 (see Figure 2).

We conducted separate Condition × Association ANOVAs for the two age groups to elucidate the sources of the three-way interaction. Analysis of data from 7-month-olds yielded a reliable main effect of Association, *F*(1, 34) = 7.17, *p* < 0.05, partial η^2^ = 0.17, and no other significant effects (Figure 2). Analysis of data from 14-month-olds yielded a reliable main effect of Condition, *F*(1, 34) = 8.80, *p* < 0.01, partial η^2^ = 0.21, reflecting longer looking overall by infants in the Speech condition (*M* = 9.84 s, SD = 4.33) vs. the Tones condition (*M* = 6.99 s, SD = 5.93), and a reliable Condition × Association interaction, *F*(1, 34) = 7.10, *p* < 0.05, partial η^2^ = 0.17. *Post hoc* comparisons (simple effects tests) revealed longer looking times during Switch trials vs. Same trials in the Speech condition, *F*(1, 34) = 8.47, *p* < 0.01, but no reliable differences between Switch and Same trials in the Tones condition, *F*(1, 34) = 0.74, ns.

To examine the hypothesis that our analysis was underpowered due to an insufficient sample size (as suggested by a reviewer), we repeated analysis of looking times from 7-month-olds, including data from 20 infants observed in a pilot experiment^2^. The outcome of the Condition × Association analysis was largely the same: a reliable main effect of Association, *F*(1, 54) = 12.97, *p* < 0.001, partial η^2^ = 0.19, and no other significant effects.

Fourteen-month-old infants, therefore, provided evidence of learning associations between moving objects and asynchronous sounds when these sounds consisted of sung speech, but not when the sounds consisted of tones matched for musical pitch, in contrast to 7-month-olds, who appeared to learn associations from both kinds of sound under tested conditions.

## Discussion

Speech and tone stimuli were matched for musical pitch, so the principal distinction between these auditory signals was the presence or absence of phonetic detail (and the presence or absence of the human voice). In the younger sample of infants, performance in the speech and tones conditions were statistically equivalent. Under the conditions we provided, therefore, there appears to be little or no advantage for speech for young infants in the associative component of word-learning. At the same time, work by Namy (Namy and Waxman, 1998; Namy, 2001) suggests that a rich referential context representative of typical of real-world adult-child interactions may be enough even at 18 (though not 26 months) to support the assignment of word like meanings to non-canonical elements, such as gestures, even though the purely associative contexts used here were not enough own their own to support such mappings.

In our younger sample of 7-month-olds, we found that there was at best only a small (and non-significant) advantage for speech relative to tones in infants’ capacity to draw associations in a simple but direct word-learning situation; by 14 months, there was a pronounced advantage for speech, even in the current task, which is focused as closely as possible on the purely associative component of word-learning. In terms of developmental sequence – though not in terms of absolute ages – the developmental trend we found might be taken as broadly consistent with the developmental trend in Namy and Waxman (1998) and Woodward and Hoyne (1999) who found an advantage for speech relative to non-linguistic stimuli at 26 and 20 months (in their respective studies) but not at 18 and 13 months. Though more research clearly needs to be done, this parallel signals that there is a genuine advantage for speech in word-learning contexts, but this advantage may accumulate relatively gradually, not as an immediate product of the potential attention-grabbing properties of the sheer acoustic stimulus of speech (which might be apparent in the absence of experience) but rather only once children have developed a small database of familiar words, and learn to integrate that database in different ways with different word-learning situations. Alternatively, advantages for speech may begin to emerge as children develop a greater awareness of the referential capacity of words, and may have been facilitated in our experiment by an increasing sensitivity to ostensive information provided by the hand as it moved the objects.

Whereas as Xu (2002) and Fulkerson and Waxman (2007) suggested that some aspects of word-learning (e.g., categorization and individuation) may be most effective when used in conjunction with a verbal label, our results suggests that other aspects of word-learning, such as the simple association between a particular label and a particular individual, may hold no special advantage for speech early in development. Why the difference? One possibility is that tasks such as categorization and individuation may be more demanding than association alone. As cognitive challenges become more demanding, the perhaps richer scaffolding provided by words may become important in allowing young children to manage cognitive loads. Recent work by MacKenzie et al. (2011) might be seen as consistent with that conjecture: infants performed better on a switch task in which novel stimuli are paired with complete (richly represented) words than a switch task in which novel stimuli are paired with sparser linguistic stimuli (such as isolated vowel or consonant sides).

At the same time, it is worth noting that association *per se* is an evolutionarily ancient process, conserved across virtually all multicellular creatures, and as such, something that emerged well before language appeared; it is also present at birth (Slater et al., 1997, 1999), and likely to be available even *in utero* (Kawai, 2010). More abstract processes, in contrast, such as tracking an object over time with reference to a sortal associated with it (Xu, 2007), may be relatively more recent phylogenetically, later developing ontogenetically (Xu and Carey, 1996), and more deeply intertwined with language, and as such may benefit more from linguistic context.

Ultimately, word-learning, like language itself, may rely on a rich interplay of mechanisms, some developed quite recently and tuned closely to speech, others ancient and available in a wide variety of cognitive domains (Hauser et al., 2002; Marcus, 2006). The associative component may ultimately be tunable to particular classes of stimuli, as our results with older infants suggest, but in the case of word-learning such tuning may itself depend on first developing a degree of familiarity with how one’s particular language represents words. An intriguing open question is how young language learners learn to incorporate associative learning into the larger project of acquiring language. Familiar arguments by Miller and Chomsky (1963) suggest that association alone is not enough to master language, but to the extent that association plays an important, and gradually refined, role in language acquisition, it is important to understand how and when association is incorporated.

Going forward, an interesting test case might be to investigate whether children learning sign language, in which words are represented differently (Rabagliati et al., 2012), and certain systems such as verb morphology tend to be more iconic than in spoken-language counterparts (Aronoff et al., 2005), would show similar developmental trends.

## Conflict of Interest Statement

The authors declare that the research was conducted in the absence of any commercial or financial relationships that could be construed as a potential conflict of interest.

## Footnotes

^1^The contrast between domain-general and domain-specific may not be a simple dichotomy. A given process could, for instance, be specialized for word-learning, for language more generally, or for, say, complex sounds in particular, or, at the opposite extreme, it might (like a concatenation operation in many programming languages) apply to any content whatsoever. For present purposes, we focus solely on understanding the domain of association in word-learning contexts, and how that changes over the course of development.

^2^The pilot experiment with 7-month-olds followed methods as described previously, but with two differences: (a) we added a post-test trial with a silent giraffe movie to test for posthabituation recovery to a completely novel stimulus, and (b) we used a habituation criterion that allowed only for brief habituation intervals (a minimum of four trials) prior to presentation of test displays. In this experiment we observed significant recovery to the novel stimulus relative to the last habituation trial, *t*(35) = 2.28, *p* < 0.05. However, a substantial portion of the infants (16 of the 36 infants tested) reached the habituation criterion after only four trials (the minimum number possible under the habituation criterion), which, we reasoned, may have been insufficient to learn the visual-auditory association. Our intuition was supported by a comparison of looking times toward Switch (*M* = 9.95 s, SD = 6.41) vs. Same (*M* = 7.03 s, SD = 3.43) displays for those 20 infants who took more than four trials to habituate; this comparison was statistically significant, *t*(19) = 2.14, *p* < 0.05. Also statistically significant was a comparison of total time to habituate by the two groups (*M* = 94.95 s, SD = 33.95 vs. *M* = 172.23 s, SD = 75.19), *t*(34) = 3.80, *p *< 0.001.
